# Seasonal variation in Swedish sarcoidosis patients with severe hypercalcemia

**DOI:** 10.1038/s41598-025-89942-w

**Published:** 2025-02-13

**Authors:** Joanna Werner, Pernilla Darlington, Natalia Rivera, Anders Eklund, Anna Smed-Sörensen, Susanna Kullberg

**Affiliations:** 1 Division of Immunology and Respiratory Medicine, Department of Medicine Solna, Karolinska Institutet, Gävlegatan 55, NB3:03, 171 76 Stockholm, Sweden; 2 Department of Respiratory Medicine, Theme Inflammation and Ageing, Karolinska University Hospital, 171 76 Stockholm, Sweden; 3 Department of Internal Medicine, Sjukhusbacken 10, Södersjukhuset, Stockholm, 118 83 Sweden; 4 Department of Clinical Science and Education, Södersjukhuset and Karolinska Institutet, Stockholm, 118 83 Sweden; 5 Department of Clinical Immunology and Transfusion Medicine, Karolinska University Hospital, Stockholm, 171 76 Sweden

**Keywords:** Biomarkers, Genetics research, Translational research

## Abstract

Sarcoidosis associated hypercalcemia (SAHC) is a challenging clinical problem as it can result in severe morbidity. Sunlight exposure and conversion of vitamin D to its active form by macrophages in granulomas have been suggested as possible causes. We aimed to disentangle mechanisms behind SAHC by investigating any associations with season, granuloma burden and lung macrophages. Patients with SAHC were identified from a local cohort. The patients were divided in two groups: mild and severe SAHC. Data on when SAHC occurred, *HLA-DRB1* alleles, bronchoalveolar lavage fluid (BALF) macrophages, extrapulmonary manifestations (EPM) and serum angiotensin-converting enzyme (s-ACE) as a marker for granuloma burden were retrieved from medical records. Out of 83 patients with SAHC, severe hypercalcemia was found in 36 patients, 75% of whom presented between May and October (*p* < 0.001). No seasonal variation was observed for patients with mild hypercalcemia. Elevated s-ACE was more common in patients with severe hypercalcemia (84% of patients), compared to 46% in the group with mild hypercalcemia (*p* < 0.001). HLA-DRB1*04 was more frequent in the group with severe hypercalcemia, compared to the mild group (67% vs. 32%, *p* < 0.01). Results support SAHC being associated with sun exposure. Risk factors to be observant of are elevated s-ACE and HLA-DRB1*04.

## Background

Sarcoidosis is an inflammatory disease of unknown origin. The formation of non-caseating granulomas is the defining characteristic of sarcoidosis and they are in 90% of patients identified in the intrathoracic lymph nodes and/or lungs^1,2^, but almost any organ can be affected. Activated macrophages can produce serum angiotensin-converting enzyme (s-ACE), which could be used as a biomarker for sarcoidosis and believed to reflect granuloma burden^3–6^.

The appearance and progression of the disease, including extrapulmonary manifestations (EPM) are heterogeneous and vary by ancestry and geography^7^, and are partly explained by individual differences in *HLA*-*DRB1* alleles^8^. The phenotype Löfgren’s syndrome (LS) is characterized by an acute onset and often comes to spontaneous resolution. In contrast, patients with non-Löfgrens syndrome (non-LS), usually have a slower debut and a more prolonged disease course^9^. EPM frequently occur in non-LS patients and associate with *HLA*-DRB1*04 but occur only rarely in LS patients^10^. The clinical importance of these manifestations varies. Sarcoidosis associated hypercalcemia (SAHC) is a challenging problem as it can cause severe morbidity and call for urgent treatment initiation, but the mechanisms underlying SAHC have so far not been very well studied. Disease-related alterations in calcium metabolism are believed to, at least partly, explain SAHC.

Vitamin D is ingested as vitamin D2 or D3, and vitamin D3 can also be converted from 7-dehydrocholesterol in the skin by sun exposure^11^. A two-step hydroxylation process, first conducted in the liver and subsequently mostly in the kidney, produces the active vitamin D. In sarcoidosis, activated macrophages can produce the enzyme 1-alpha-hydroxylase that converts 25(OH) vitamin D3 to the active form of vitamin D^12–14^. Excessive amounts of active vitamin D increase calcium uptake from the intestines and enhance bone resorption, leading to hypercalcemia^15^.

We recently reported an association with SAHC, *HLA*-DRB1*04, s-ACE and EPM, especially in patients with severe SAHC^16^, supporting the hypothesis of activated macrophages influencing calcium levels in sarcoidosis^15^. Previous studies on the impact of sun exposure on SAHC consist mostly of small cohorts and case reports and have yielded conflicting results^17–22^.

Hypothesizing that SAHC is influenced not only by macrophage activation but also by sun exposure, we examined seasonal variation of SAHC in Swedish non-LS sarcoidosis patients.

## Materials and methods

### Study subjects

From a local register of sarcoidosis patients at Karolinska University Hospital, non-LS patients with SAHC collected between 1994 and 2022 were identified. In brief, the register includes clinical and laboratory data from patients with sarcoidosis. The majority of patients were included in a previous study where cohort details are available^16^. All included patients were diagnosed with sarcoidosis using the criteria outlined by the World Association of Sarcoidosis and other Granulomatous Disorders (WASOG)^23^. They were not taking vitamin D or calcium supplementation, or had any other known causes for hypercalcemia, such as hyperparathyroidism. The study was approved by the Regional Ethical Review Board in Stockholm (2005/1031-31/2, 2018/2425-32). Information about the study was given both orally and written, and informed consent according to the declaration of Helsinki was signed by all subjects and/or their legal guardian(s). All methods were carried out in accordance with relevant guidelines and regulations.

## Parameters

Hypercalcemia was defined as serum Ca^2+^ (s-Ca^2+^) > 1.33 mmol/L (reference value 1.15–1.33 mmol/L) for most patients, but due to changes in reference values over the study period, as s-Ca^2+^>1.34 mmol/L for two patients and > 1.35 mmol/L for twelve patients. We previously showed that especially s-Ca^2+^>1.4 mmol/L leads to elevated p-creatinine and thus is of clinical impact, therefore this group was analyzed separately and referred to as severe SAHC^16^. Seasonal variation was analyzed by dividing the year into two time periods, May to October (light period) and November to April (dark period). This division was made with reference to sun exposure levels in Sweden, and the assumption of a slight delay in developing SAHC after sun exposure, previously reported by Taylor et al.^24^. We also divided the year into three-month periods (Jan-Mar, Apr-Jun, July-Sept and Oct-Dec). Data on sex, age, chest x-ray according to Scadding, s-ACE, EPM, *HLA*-*DRB1* alleles, bronchoalveolar lavage fluid (BALF) macrophages (concentration x10^6^/L, and percentage of total number of cells) and which month hypercalcemia developed for the first time, were retrieved from the local register and medical records. EPM was defined as a positive biopsy from the affected organ or obvious symptoms/assessment from a specialist in the area. Whether the EPM were active with an ongoing inflammation or represented fibrotic remnants from previous inflammation were not assessed. Manifestations associated with hypercalcemia i.e. hypercalciuria and renal stones were not regarded as EPM.

### Statistical analysis

Differences between groups were analyzed with Fisher’s exact test or Chi-Square where appropriate. Chi-square goodness of fit was used to analyze seasonal variation of SAHC expecting it to be equally distributed. To minimize the risk of type 1 error due to multiple testing of the 13 different alleles, a Bonferroni correction p-value was set to determine statistical significance (*p* < 0.05/13 = *p* < 0.0038). P-values are presented uncorrected. Mann-Whitney U test was used to analyze BALF macrophage counts and percentages, in the mild and severe SAHC groups, respectively. A nominal p-value of < 0.05 was regarded as significant. Statistical analyses were performed with Graph Pad Prism 8 (Graph Pad Prism 8 (GraphPad Software Inc., San Diego, CA, USA and Jamovi (The jamovi project (2023). *jamovi* (Version 2.4.8.0) [macOS Sonoma, version 14.3]. Retrieved from https://www.jamovi.org).

## Results

Eighty-three patients with SAHC were identified, 47 of which had mild SAHC and 36 had severe SAHC, see Table 1.

**Table 1:** Clinical characteristics of patients with SAHC. Patients on angiotensin-converting enzyme (ACE) inhibitor treatment were excluded from analysis of ACE (1 and 4 from the mild and severe group, respectively). SAHC = sarcoidosis associated hypercalcemia, EPM = extrapulmonary manifestations. HLA-DRB1*04 refers to patients carrying this allele. P-value denotes comparison between mild and severe SAHC, Ns = non-significant.

	All patients *n* = 83	Mild SAHC (s-Ca^2+^ 1.33–1.4) *n* = 47	Severe SAHC (s-Ca^2+^>1.4) *n* = 36	*p*-value
Men n (%)	52 (63)	29 (62)	23 (64)	ns
Age, mean	47	45	49,5	ns
Scadding stage 0-IV, n	6/20/46/7/4	3/13/24/3/4	3/7/22/4/0	ns
Elevated s-ACE, n (%)	48 (62)	21 (46)	27 (84)	*p* = 0.0003
EPM, n (%)	65 (78)	36 (77)	29 (81)	ns
*HLA*-DRB1*04 n (%)	39 (47)	15 (32)	24 (67)	*p* = 0.002
Median macrophage count x10^6^/L (25th -75th percentile)	121 (97–172)	135 (97–224)	117 (99 − 47)	ns
Median macrophage percentage (25th -75th percentile)	65 (53–74	64 (60–75)	65 (53–72)	ns

**Fig. 1:**
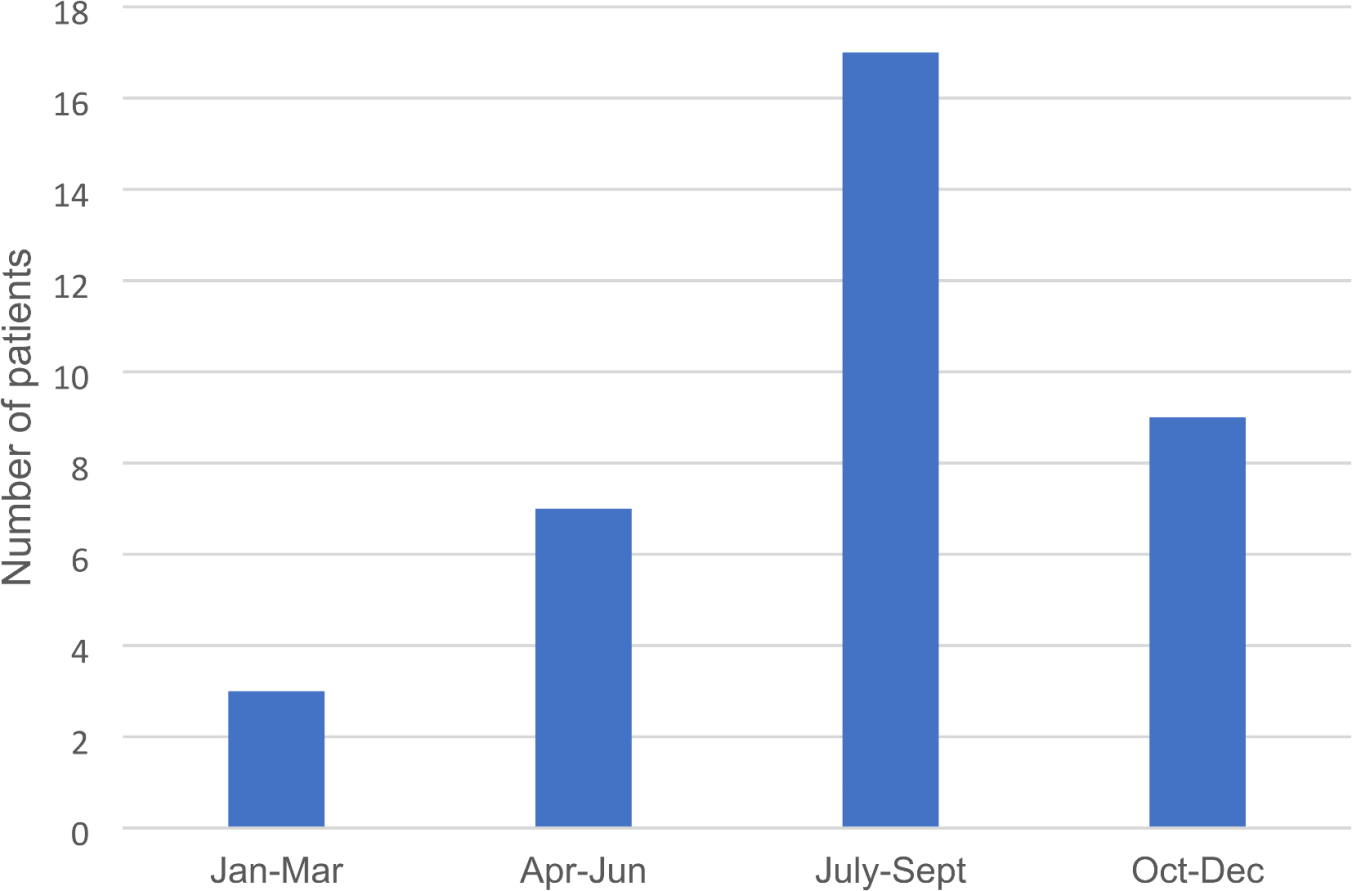
Seasonal variation in patients with s-Ca2^+^>1.4 mmol/L. Patients stratified in quarters of the year, based on when they were diagnosed with s-Ca2^+^>1.4 mmol/L, i.e. severe sarcoidosis associated hypercalcemia. Most common was between July and September, *p* < 0.01.

Out of 36 patients with severe SAHC, 27 (75%, *p* < 0.001) presented with SAHC between May and October. However, between July and September, 17 of 36 patients (47%, *p* < 0.01) were diagnosed with severe SAHC, see Fig. 1. By contrast, no seasonal variation was found in patients with mild SAHC.

**Fig. 2:**
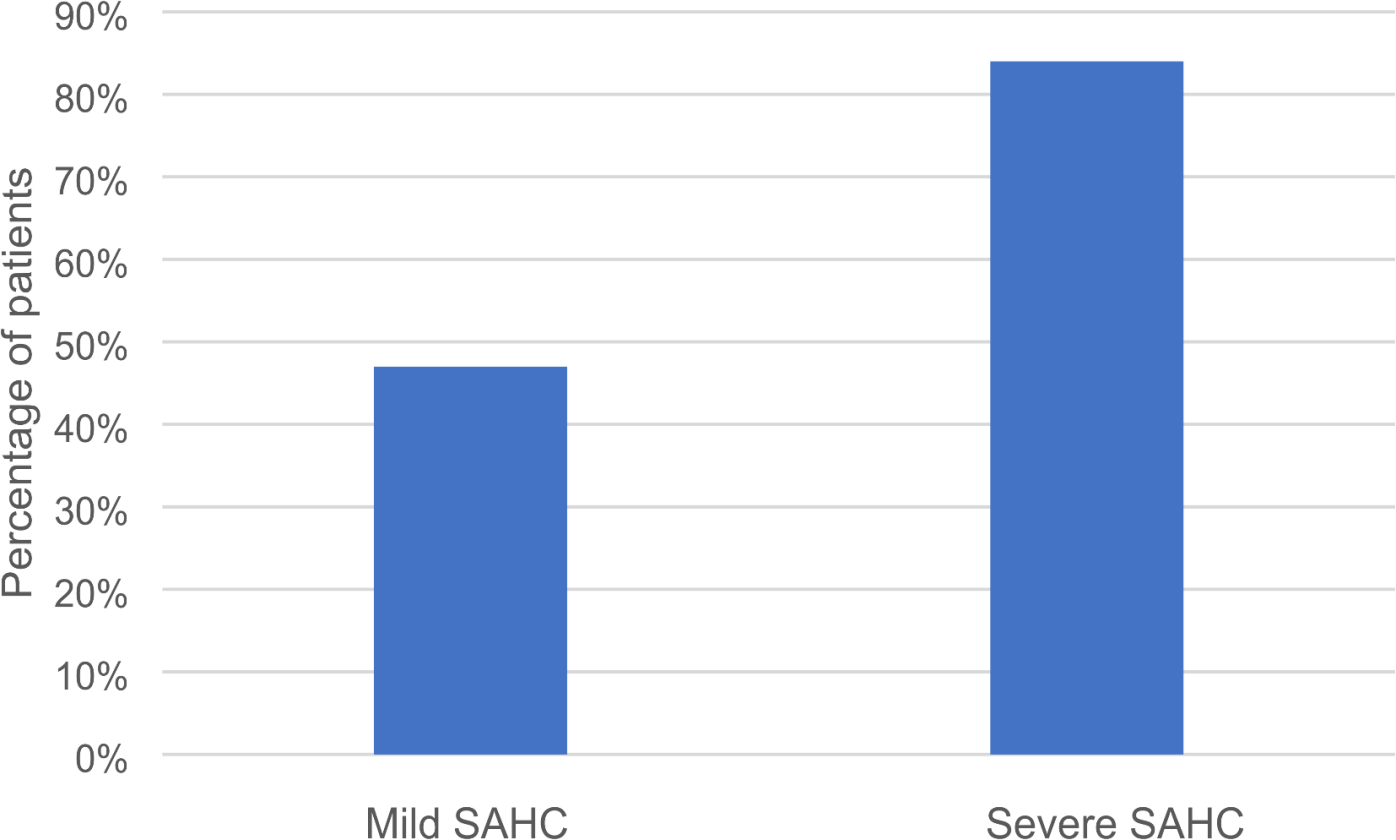
Percentage of patients in mild and severe SAHC groups with elevated s-ACE. Elevated s-ACE was most common in patients with severe sarcoidosis associated hypercalcemia (SAHC), *p* < 0.001. Mild refers to s-Ca^2+^ 1.33–1.4 and severe s-Ca^2+^>1.4.

**Fig. 3:**
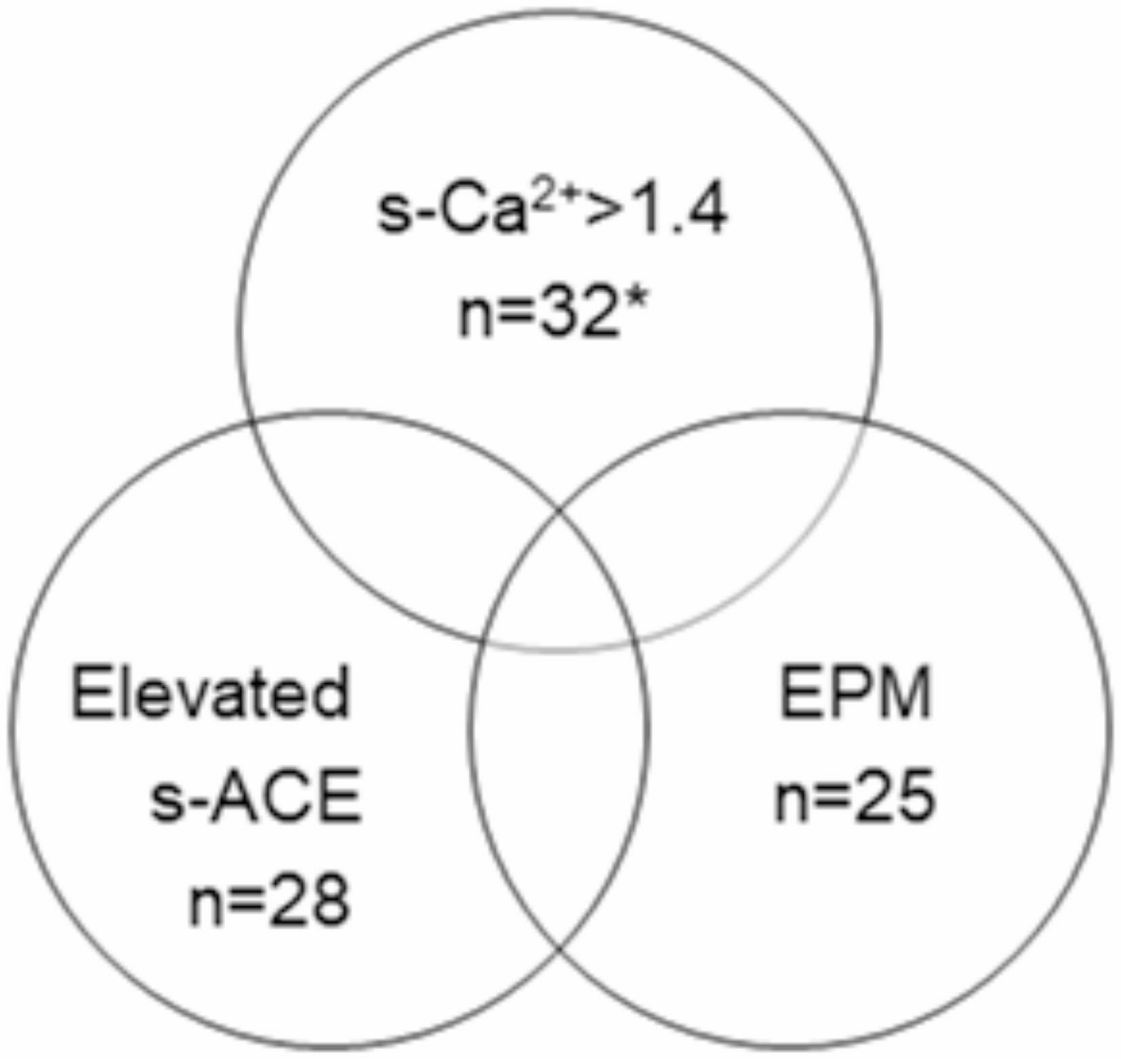
Association between severe SAHC, EPM and elevated s-ACE. Severe sarcoidosis associated hypercalcemia (SAHC) is denoted by s-Ca^2+^>1.4 mmol/L. EPM = extrapulmonary manifestations, s-ACE = serum angiotensin-converting enzyme. *Four patients with severe SAHC were excluded due to ACE inhibitor treatment.

S-ACE was frequently elevated in patients with severe SAHC (84%) compared to patients with mild SAHC (46%, *p* < 0.001), shown in Fig. 2. The presence of EPM was more common in patients with severe SAHC, 81% compared to 77% in patients with mild SAHC, however the difference was non-significant. *HLA*-DRB1*04 carriage was associated with severe SAHC (67%) compared to patients with mild SAHC (32%, *p* = 0.002 i.e. significant according to Bonferroni correction), see Table 1. All patients with severe SAHC had either elevated s-ACE and/or an EPM, see Fig. 3.

BALF macrophage counts and percentages were available from 53% (*n* = 25) and 64% (*n* = 30), respectively, of patients with mild SAHC. Median macrophage count was 135 × 10^6^/L (25th -75th percentile, 97–224) and percentage 64% (60–75) in patients with mild SAHC. In patients with severe SAHC, macrophage counts and percentages were available from 39% (*n* = 14) and 56% (*n* = 20), respectively, of patients. Median macrophage count was 117 × 10^6^/L (25th -75th percentile, 99–147) and for percentage 65% (53–72) in patients with severe SAHC. For patients with mild SAHC, the median time between BAL and SAHC was 28 days compared to 65 days for patients with severe SAHC. However, variability was high, in some cases several years between BAL and SAHC. We observed no correlations between SAHC and BALF macrophage data.

## Discussion

To our knowledge, this is the largest study concerning the seasonal variation of SAHC. Severe SAHC in Swedish sarcoidosis patients was most prevalent during the summer months. Strong correlations between SAHC and s-ACE and *HLA*-DRB1*04, previously described using a smaller cohort^16^, was supported here. As the majority of patients, especially those with severe SAHC, were diagnosed with elevated s-ACE, indicating a large granuloma burden, we hypothesized that upregulated activation of vitamin D within the granulomas resulted in high calcium levels. Patients with severe SAHC more often had EPM than patients with mild SAHC, however the difference was small and non-significant. This may have several explanations. Although patients at our clinic underwent extensive blood screening for EPM, they were not routinely screened with radiographic methods outside the thoracic cage, hence EPM may be underestimated. Also, we lack data on activity of EPM at the time for SAHC. Thus, considering the described limitations in EPM assessment, we believe s-ACE may be a better marker for granuloma burden in this study,

Earlier studies on seasonal variation in SAHC have yielded somewhat conflicting results. Several case reports and small studies have shown a seasonal variation in SAHC^17–20^. Findings indicate that SAHC is more common in white sarcoidosis patients than in patients of African ancestry^1,24^. This might explain the different results found by James et al. (1976), who showed that hypercalcemia was more common in patients in London and Lisbon than in Los Angeles, and Sharma et al. (2000) who concluded that sunlight exposure did not have a clinical influence in hypercalcemia^21,22^. However, the majority of patients in the Los Angeles study were of African ancestry^21^, and thus probably more resistant to sun exposure effects on vitamin D. Also, a seasonal variation in SAHC may be explained by the more significant differences in sunlight exposure through a calendar year in northern latitudes.

Studying relationships between seasonal variability in light wave lengths and SAHC, Taylor et al.^24^ found that conversion of vitamin D to its active form was most effective, from March to November in the northern temperature zone, and that hypercalcemia in sarcoidosis increased between May and November, and was highest in June to August. Our results are in line with Taylor et al. (1963) and are also consistent with sun exposure in Stockholm, according to charts from the Swedish Meteorological and Hydrological Institute^25^. We showed an increase in cases with severe SAHC between May to October, peaking between July and September. This supports our hypothesis that sun exposure has an important role in SAHC, as well as highlighting the importance of conversion of vitamin D2 to vitamin D3. An upregulated activation process from sarcoid granulomas, further activates vitamin D, leading to SAHC.

Despite our findings supporting a role for activated macrophages in SAHC, we did not find any significant correlations with either percentages or concentrations of BALF macrophages and calcium levels. This may have several explanations, for instance the time of BAL did not always correspond to the date when SAHC emerged. Also, the number of patients with BALF macrophage data was limited resulting in poor statistical power. However, although the s-ACE levels have mainly been related to stage II and III, the levels are higher in patients with EPM than in patients with isolated pulmonary involvement^6,26^. Furthermore, it is not certain that alveolar and epithelioid macrophages within granulomas are related. Sarcoidosis mononuclear phagocytes disclose differential maturation status between compartments^27,28^ and dermal macrophages are likely to respond to UVA exposure^29^.

Thus, it is tempting to speculate that alveolar macrophages might have a lesser degree of activation compared to extrapulmonary macrophages and/or that release of mediators does not reach the systemic circulation to such an extent that it has an impact on SAHC. Finally, not all severe SAHC cases were associated with the light season, indicating the importance of other variables such as high granuloma burden and excessive dietary intake of calcium/vitamin D, to which sunlight may have an additive effect.

.

There are limitations in our study. One major limitation is the retrospective study design, and thus the risk of biases associated with retrospective analyses. Another limitation is that we lacked dietary information on calcium and vitamin D, and individual degree of sun exposure, both of which can confound our interpretations. From a clinical perspective, we know that the majority of patients in our study are of Nordic descent, but not having data on ancestry, is a shortcoming. The already mentioned limitation about not actively screening for EPM, is to a certain degree balanced by a very systematic work-up in a broad sense of all patients, making a major impact of EPM rather unlikely. However, a functional impact cannot be totally excluded. Another limitation is that we did not have information about ACE gene polymorphism, which is linked to s-ACE levels, and thus may have influenced our results^6^. Also, another suggested mechanism is the production of parathyroid hormone-related peptide (PTHrp) in the granulomas causing SAHC through enhanced bone resorption and tubular calcium reabsorption^30^.

Our study has some major strengths including being, to our knowledge, the largest conducted study on seasonal variation in SAHC patients^17–20,31,32^. The sarcoidosis diagnosis is validated by several clinicians who are specialists in respiratory medicine and the population is homogenous with a majority of European white ancestry individuals, specifically Nordic descent, all with non-LS and characterized both geno- and phenotypically in a systematic and consistent way.

## Conclusion

To conclude, findings from this study indicate that severe SAHC in Swedish sarcoidosis patients is associated with sun exposure, elevated s-ACE and *HLA*-DRB1*04. Accordingly, it is judicious to advise patients with elevated s-ACE and HLA-DRB1*04 carriage to avoid excessive exposure to the sunlight as well as to monitor them for hypercalcemia especially during the light period of the year. As we now increase our dataset by including measures of vitamin D, sun exposure and dietary habits, we hope to be able to further predict which patients are at risk of developing SAHC due to sunlight exposure.

## Data Availability

The datasets used and/or analyzed during the current study are available from the corresponding author on reasonable request.
